# Emergent bimodal firing patterns implement different encoding strategies during gamma-band oscillations

**DOI:** 10.3389/fncom.2013.00018

**Published:** 2013-03-26

**Authors:** B. Sancristóbal, R. Vicente, J. M. Sancho, J. Garcia-Ojalvo

**Affiliations:** ^1^Department of Experimental and Health Sciences, Barcelona Biomedical Research Park, Universitat Pompeu FabraBarcelona, Spain; ^2^Departament de Física i Enginyeria Nuclear, Universitat Politècnica de CatalunyaTerrassa, Spain; ^3^Department of Neurophysiology, Max-Planck Institute for Brain ResearchFrankfurt am Main, Germany; ^4^Frankfurt Institute for Advanced StudiesFrankfurt am Main, Germany; ^5^Departament d'Estructura i Constituents de la Matèria, Universitat de BarcelonaBarcelona, Spain

**Keywords:** gamma oscillations, local field potential, bimodal, coding, bursting

## Abstract

Upon sensory stimulation, primary cortical areas readily engage in narrow-band rhythmic activity between 30 and 90 Hz, the so-called gamma oscillations. Here we show that, when embedded in a balanced network, type-I excitable neurons entrained to the collective rhythm show a discontinuity in their firing-rates between a slow and a fast spiking mode. This jump in the spiking frequencies is characteristic to type II neurons, but is not present in the frequency-current curve (*f-I* curve) of isolated type I neurons. Therefore, this rate bimodality arises as an emerging network property in type I population models. We have studied the mechanisms underlying the generation of these two firing modes, in order to reproduce the spiking activity of *in vivo* cortical recordings, which is known to be highly irregular and sparse. We have also analyzed the relation between afferent inputs and the single unit activity, and between the latter and the local field potential (LFP) phase, in order to establish how the collective dynamics modulates the spiking activity of the individual neurons. Our results reveal that the inhibitory-excitatory balance allows two encoding mechanisms, for input rate variations and LFP phase, to coexist within the network.

## 1. Introduction

The type and distribution of ionic channels across the membrane of a neuron determine its excitability behavior. This behavior can be experimentally tested by injecting a pulsed, constant, or ramp current. In the latter case one observes how the membrane potential changes as the input amplitude increases, eventually switching from a resting to an oscillating (tonic firing) state. Mathematically, this transition is described as a bifurcation of the membrane potential from a stable fixed point to a periodic orbit.

Depending on how the firing frequency within the oscillatory regime behaves near the bifurcation, neural excitability can be classified into *type I* or *type II* (Rinzel and Ermentrout, 1989). In type I excitability, the frequency increases continuously from zero as the bifurcation is crossed. Type II neurons, on the other hand, exhibit a discontinuous jump in frequency as the tonic regime is entered, and their range of firing frequencies is quite narrow compared to type I neurons, which can achieve arbitrarily low frequencies (Izhikevich, 2007). These different behaviors are associated with distinct bifurcations. In type I excitability, the periodic orbit emerges due to the collision of a stable fixed point (the resting state) and an unstable equilibrium point, occurring on top of an invariant circle (SNIC bifurcation). Type II excitability, on the other hand, can arise in three different ways: via a subcritical or a supercritical Hopf bifurcation, or through a saddle-node bifurcation *outside* the invariant circle (Rué and Garcia-Ojalvo, 2011). The integrate-and-fire and conductance-based models used in the literature to describe cortical networks are usually of type I (Wang and Buzsáki, 1996; El Boustani et al., 2007).

The phase response can also be used as a criterion to distinguish between excitability classes: when operating in a tonic regime, type I neurons always advance their phase (defined with respect to their spiking period) when perturbed by a brief depolarizing pulse, while type II neurons can either advance or delay their phase depending on the instant of perturbation relative to the period of oscillation (Hansel et al., 1995). Within a network, the input pulses come from other neurons, and therefore synchronized periodic spiking might lead to lower or higher frequency rhythms depending on the type of neuronal excitability. However, high spike synchrony and periodic firing patterns are rarely seen *in vivo*; in fact the coefficient of variation of interspike intervals in the cortical neurons is typically larger than 1 (Shinomoto et al., 1999, 2005). Consequently, the rhythm of the neural population must emerge from the recurrent synaptic activity in the network, rather than from spike-to-spike synchrony. It has been experimentally observed (Softky and Koch, 1993) that the high synaptic bombardment acting upon cortical neurons is far from being constant, leading to stochastic fluctuations that affect these neurons *in vivo*. Thus, neither the single-neuron frequency-current curve (*f-I* curve) nor the phase response can perfectly describe the behavior of single neurons when they are embedded in a network.

Cortical oscillations thus arise as a collective phenomenon, which does not require individual-neuron firings to be oscillatory themselves. These global oscillations are discernible in averaged population activities such as the local field potential (LFP), whose troughs (or peaks, depending on the relative position of the recording electrode and the generating current dipoles) correspond to the minima of the synaptic inhibitory flow. In these temporal windows neurons are more likely to spike, producing an increase in the excitatory synaptic current, followed by a new burst of inhibition. These oscillations, at frequencies in the beta (12–30 Hz) and gamma (30–90 Hz) ranges, are experimentally seen in the cortex upon sensory stimulation (Buzsáki and Draguhn, 2004). Specifically, gamma-band synchronization has received special consideration, as it is hypothesized to be a mechanism for the dynamic generation of functional cell assemblies and for the flexible communication between brain areas [for a review, see Singer (1999) and Fries (2009)].

Here we study the generation of gamma-band (30–90 Hz) oscillations that occur without spike-to-spike synchrony [known as synchronous irregular dynamics (van Vreeswijk and Sompolinsky, 1996; Brunel, 2000; Hansel and Mato, 2003)], and how these rhythms affect single unit activity. Specifically, we consider networks of type I excitatory and inhibitory neurons described by conductance-based models and stimulated by slowly varying excitatory inputs, and study the emergent firing patterns of the neurons once embedded in a balanced network. Our results show that the synchronous irregular state giving rise to gamma-band oscillations is composed of two firing modes: a high-frequency (or bursty) regime and a low-frequency regime separated by a gap of quasi-forbidden firing frequencies. We also study the relationship between this firing behavior and the input: the fast spiking mode encodes for the stimulus rate, whereas the existence of a slow spiking mode allows for a phase code to appear.

## 2. Methods

### 2.1. Description of the neuronal models

The dynamical equation for the neuronal membrane voltage is given by a conductance-based model:

(1) $$\begin{array}{l} C_{m}\frac{dV}{dt}=-g_{K}n^{4}(V-V_{K})-g_{Na}m^{3}h(V-V_{Na})\\ \;-g_{L}(V-V_{L})+I_{\text{syn}} \end{array}$$

where *g*_*K*_, *g*_*Na*_, and *g*_*L*_ are the maximum conductances for the potassium, sodium and the leak current, respectively, and *I*_syn_ is the synaptic current coming from the neighboring neurons impinging on one neuronal cell. The dynamics of the potassium and sodium channels is represented by the time-varying probabilities that a channel is open:

$$\frac{dx}{dt}=\phi \left[\alpha_{x}(V)(1-x)-\beta_{x}(V)x\right]\text{​},$$

where *x* stands for *n* in the case of the potassium current, and for *m* and *h* in the case of the sodium current. α(*V*) and β(*V*) are voltage-dependent rate constants, and φ is the temperature factor, defined by φ = 3^(*T*−6.3)/10^, where *T* is measured in degrees Celsius.

The parameter values used throughout this study are those of Ref. Gutfreund et al. (1995): *g*_*K*_ = 4.74 μS, *g*_*Na*_ = 12.5 μS, and *g*_*L*_ = 0.025 μS. The reversal potentials of the different channels are *V*_*K*_ = −80 mV, *V*_*Na*_ = 40 mV, and *V*_*L*_ = −65 mV, and the membrane capacitance is *C*_*m*_ = 0.25 nF (0.125 nF) for the excitatory (inhibitory)neurons. The leak conductance defines an effective membrane time constant for the isolated neuron according to the expression τ = *C*_*m*_/*g*_*L*_, which is taken to be 10 ms for the excitatory neurons and 5 ms for the inhibitory neurons. The temperature factor φ is set to 21, which corresponds to *T* = 34°C. These parameter values lead to type I excitability.

The rate functions α and β for each gating variable are:

$$\begin{array}{l} \alpha_{n}(V)=0.01\frac{V+20}{1-e^{-(V+20)/10}}\\ \beta_{n}(V)=0.125e^{-(V+30)/80} \end{array}$$

for the gating variable *n*,

$$\begin{array}{l} \alpha_{m}(V)=0.1\frac{V+16}{1-e^{-(V+16)/10}}\\ \beta_{m}(V)=4e^{-(V+41)/18} \end{array}$$

for the gating variable *m*, and

$$\begin{array}{l} \alpha_{h}(V)=0.07e^{-(V+30)/20}\\ \beta_{h}(V)=\frac{1.0}{1+e^{-V/10}} \end{array}$$

for the gating variable *h*. Due to the rapid activation of *m* we replace it by its steady-state value $m_{\infty }=\frac{\alpha_{m}}{\alpha_{m}+\beta_{m}}$.

### 2.2. Description of the network model

We consider a network composed of 2000 neurons, 80% of which are excitatory while the remaining 20% are inhibitory (Soriano et al., 2008). All connections between cells are chemical synapses—no gap junctions are considered—and each neuron connects randomly with 200 other neurons, on average, belonging to both populations. Therefore, no architecture is imposed on the connectivity. We have also introduced a synaptic delay in the transmission of the action potential between neighboring neurons, taken from a gamma distribution of mean 2 ms and variance 4 ms^2^. The synaptic current is described using again a conductance-based formalism:

$$I_{\text{syn}}=g_{\text{syn}}(t)(V(t)-E_{\text{syn}}),$$

where *g*_syn_ is the synaptic conductance and *E*_syn_ is the reversal potential of the synapse. For *E*_syn_ greater than the resting potential *V*_rest_ the synapse is depolarizing, i.e., *excitatory*, otherwise it is hyperpolarizing, i.e., *inhibitory*. We consider two temporal time constants, τ_*d*_ and τ_*r*_ (decay and rise synaptic time, respectively, see Table 1), for the dynamics of the synaptic conductance, which is calculated by

$$g_{\text{syn}}(t)=\frac{{g^{\prime }}_{\text{syn}}}{\tau_{d}-\tau_{r}}\left[e^{\frac{-t-t_{j}}{\tau_{d}}}-e^{\frac{-t-t_{j}}{\tau_{r}}}\right]\text{​},$$

where *g*′_syn_, shown in Table 2, is tuned in order to obtain a balance between excitation and inhibition, given the *f-I* relation.

**Table 1:** **Synaptic time constants and reversal synaptic potential values**.

**Synapse**	**τ**_**r**_ **(ms)**	**τ**_**d**_ **(ms)**	***E***_**syn**_ **(mV)**
AMPA	0.5	2	0
GABA	2	5	−70

The equilibrium potential at I_syn_ = 0 for Equation (1) is V_rest_ = −65 mV.

**Table 2:** **Synaptic conductances, *g*′_syn_, for all the possible connections**.

**Synapse**	**Conductance on inhibitory neurons (nS)**	**Conductance on excitatory neurons (nS)**
GABA	240	240
Recurrent AMPA	2.5	2.5
External AMPA	3.2	3.2

We have chosen the maximal conductances, *g*′_syn_, to maintain the postsynaptic potential (PSP) amplitudes within physiological ranges: the excitatory PSP in the range from 0.42 to 0.83 mV, and the inhibitory PSP from 1.54 to 1.88 mV.

Additionally, all neurons receive an heterogeneous Poisson train of excitatory presynaptic potentials with a mean event rate that varies following an Ornstein–Uhlenbeck process. This incoming external current mimics the direct input from any other area external to the network considered here. The instantaneous rate, λ(*t*), of the external excitatory train of spikes is generated according to an Ornstein–Uhlenbeck process as considered in Mazzoni et al. (2008):

$$\frac{d\lambda }{dt}=-\lambda (t)+\sigma (t)\left(\sqrt{\frac{2}{\tau }}\right)\eta (t),$$

where σ(*t*) is the standard deviation of the noisy process and is set to 0.6 spikes/s. τ is set to 16 ms, leading to a 1/*f* power spectrum for the λ time series that is flat up to a cut-off frequency $f=\frac{1}{2\pi \tau }=9.9\;Hz$. η(*t*) is a Gaussian white noise.

**Figure 1:**
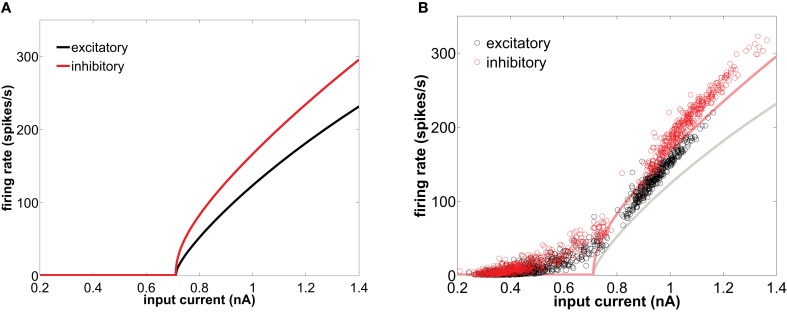
**(A)** *f-I* curve of type I conductance-based neurons in isolation, obtained with the XPPAUT software (Ermentrout, 2002). **(B)** Equivalent *f-I* curve for neurons embedded in the network (circles) representing the instantaneous firing-rate versus the net synaptic current averaged over the corresponding inter-spike interval, for an excitatory (black) and inhibitory (red) neuron. In that panel the single-neuron *f-I* curves are also shown in solid lines for comparison. The network is excited by a train of spikes of rate 8500 spikes/s.

Excitatory synapses outnumber inhibitory ones, and yet the brain avoids epileptic states because inhibition is able to balance excitation, and thus neurons remain below threshold, firing only occasionally. Inhibitory neurons have higher firing-rates than excitatory neurons for any given input current, as can be seen in the *f-I* curve of Figure 1. Additionally, GABAergic (inhibitory) synapses are stronger than glutamatergic AMPA (excitatory) synapses to compensate their relative small number (Markram et al., 2004).

### 2.3. Numerical simulations

The model has been integrated using the Heun algorithm (Garcia-Ojalvo and Sancho, 1999), with a time step of 0.05 ms. All the simulations represent 3 s of activity and the connectivity, initial conditions, and noise realization were varied from trial to trial.

### 2.4. Model of LFP

We quantify the activity of the network in different ways. At the single-neuron level we consider the instantaneous firing-rate as a measure of the individual spiking dynamics. At the population level we use two observables: the time-resolved average firing-rate of the whole neuronal population (defined as the total number of spikes per unit time in the population divided by the number of neurons), and the LFP, computed as the sum of the absolute values of the excitatory and inhibitory synaptic currents acting upon the excitatory neurons, averaged over this population (Mazzoni et al., 2008; Buzsáki et al., 2012):

(2) $$\text{LFP}=R_{e}\langle \left|I_{\text{AMPA}}|+|I_{\text{GABA}}\right|\rangle \text{​},$$

Here 〈…〉 denotes an average over all excitatory neurons (Berens et al., 2010). The term *I*_AMPA_ accounts for both the external excitatory heterogeneous Poisson spike train and the recurrent excitatory synaptic current due to network connectivity, while *I*_GABA_ corresponds to the recurrent inhibitory synaptic current. *R*_*e*_ represents the resistance of a typical electrode used for extracellular measurements, here chosen to be 1 MΩ. In Figure 9 the LFP was filtered with a 4th order Butterworth bandpass filter using MATLAB function *filter.m*.

### 2.5. Spike triggered average

We have calculated the spike triggered average (STA) of the LFP and of the inhibitory synaptic current impinging on the neurons. For the considered spikes we have registered these signals during a time window starting 50 ms prior to the spike and ending 20 ms after it, and computed the mean across the total number of action potentials.

### 2.6. Computation of power spectra

The LFP power spectrum was estimated using the multitaper method (Thomson, 1982) commonly used to reduce the variance of the spectra of recorded signals, which are usually very noisy. This estimator was implemented in Chronux 2.10 (Bokil et al., 2010). The multitapered power spectrum, *S*(*f*), is the average of the power spectrum of the LFP signal multiplied by *K* orthogonal Slepian functions (in our case *K* = 5), and further averaged over *N* = 20 trials:

(3) $$S(f)=\frac{1}{N}\sum_{n=1}^{N}s_{n}(f)=\frac{1}{N}\sum_{n=1}^{N}\left(\frac{1}{K}\sum_{k=1}^{K}|\;{\tilde{\text{LFP}}}_{n,k}(f){|}^{2}\right)\text{​}.$$

Here ${\tilde{\text{LFP}}}_{n,k}(f)$ is the discrete Fourier transform of the LFP(*t*) signal of the *n*-th trial, multiplied by the *k*-th Slepian function (or taper). We have considered data segments within a 500-ms sliding time window with an overlap of 50 ms, padded with zeros up to a length of 512 in order to obtain an increased sampling rate in the frequency domain. The resolution bandwidth is thus ±6 Hz. The firing-rate power spectra are also obtained by the multitaper algorithm, with the same sliding time window, overlap, and padding. In this case the average instantaneous firing-rate is obtained from an histogram of the spiking times, with a 1-ms bin. All histograms and power spectra are averaged over 20 trials.

## 3. Results

An adequate neural network model should reproduce several experimentally observed features of *in vivo* cortical oscillations: a prominent peak of the LFP power spectrum in the gamma-band resulting from external stimulation, irregular individual firing at frequencies lower than the gamma rhythm, and partial phase locking of individual spikes to the gamma cycle. We show in what follows that the neural network described above is able to reproduce these features.

### 3.1. Firing-rate of network-embedded type I neurons

In this section we study how the network shapes the firing patterns of the single neuron components. Before studying the firing modes of the cells when embedded in a network, we first characterize their behavior as single cells. When isolated, a neuron displays a characteristic transition to the tonic firing regime as the input current is gradually increased (solid lines in Figure 1). Type I integrator cells can achieve arbitrary low spiking frequencies. This behavior is a consequence of the saddle-node bifurcation on an invariant circle that this type of neuron undergoes with increasing injected current. For the same current, inhibitory cells (with a smaller membrane time constant) fire with a higher rate. However, both excitatory and inhibitory cells have the same spiking threshold.

The behavior of the firing-rate under constant injection current, shown in Figure 1A for adiabatically increasing values of the current, is not suited for characterizing the behavior of a neuron within a network. When embedded in a population, neurons are subject to synaptic input that fluctuates in time. Even when the firing of the presynaptic neurons projecting to a given postsynaptic cell is uncorrelated between them, the total input current is Gaussian distributed (Roxin et al., 2011). This input is the sum of the synaptic currents coming from all possible sources, which include recurrent excitation and inhibition (generated by the network itself), and afferent pathways terminating onto the population (represented in the model by a Poisson-distributed external train of spikes). The net synaptic current impinging on a neuron has a nearly stationary average value and fluctuates rhythmically around it (due to the network-induced gamma oscillations), crossing randomly the threshold and leading to an irregular individual firing-rate. Here, we capitalize on the natural variability of such recurrent and external synaptic drive to explore the effective *f-I* curve of neurons once embedded in a balanced recurrent network. Figure 1B (circles) represents the *f-I* response curve of type I neurons when embedded within a population. Given the above-mentioned fluctuating character of the synaptic current received by the neurons within the network, the *f-I* response is quantified here in terms of the interval between two consecutive spikes (interspike interval, or ISI) as a function of the mean synaptic current received by the neuron within that interval. This quantity is plotted in Figure 1B (circles), comparing its behavior with the corresponding *f-I* curves of the isolated neurons (solid lines). Note, however, the limitations of this comparison, as the mean synaptic current over an ISI differs from the actual fluctuating input received by a cell, specially for long periods. It is precisely in the range of low frequencies where both curves differ most. For instance, the firing-rate in the resting state region departs from zero close to the bifurcation, due to the appearance of noise-induced spikes throughout the network.

**Figure 2:**
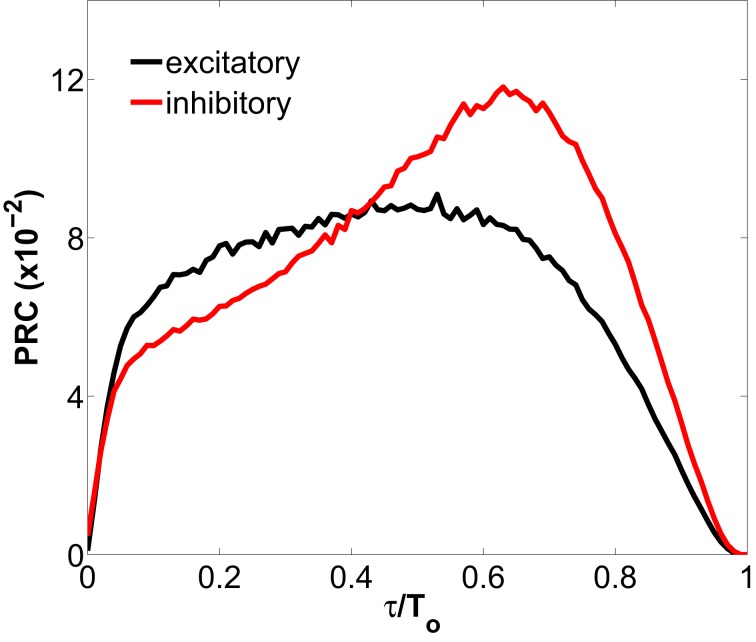
**Phase response curve for a type I excitatory and inhibitory neuron representing the phase change in the spiking response of the neuron to a depolarizing pulse, as a function of the phase of the input (normalized to the spiking period *T*_0_ of the neuron).** The excitatory (inhibitory) neuron is spiking tonically at an unperturbed period of 8.09 ms (6.00 ms), and receives the injection of a pulse with amplitude 1.0 nA and duration 0.2 ms. The PRCs, obtained with the XPPAUT software (Ermentrout, 2002), are defined as 1 − *T*(τ)/*T*_*o*_, where *T*(τ) is the period after perturbing at time τ.

Differences between the firing-rate of a network-embedded neuron and its characteristic *f-I* curve in isolation also exist in the tonic regime, as can be seen in Figure 1B. We can understand this from the fact that, due to the synaptic bombardment, neurons within a network are persistently perturbed, and consequently their firing-rates are altered. The characteristic phase response curve (PRC) of type I neurons (Figure 2) reveals a phase advance for any perturbation time within a period. Thus, when a type I neuron has just spiked and is excited again by a presynaptic potential its period is reduced (and thus its firing-rate is increased). This explains why the firing-rate of type I neurons embedded in a network, and thus continually perturbed by presynaptic activation, is above the *f-I* curve of an isolated neuron with tonic firing.

It is worth noting that the firing-rate discontinuity shown in Figure 1B (circles) is an emergent network property. Within a network, type I neurons display two clearly defined groups of inter-spike intervals, in a sort of population-level bimodality that does not exist for individual neurons in isolation.

### 3.2. Network oscillations

**Figure 3:**
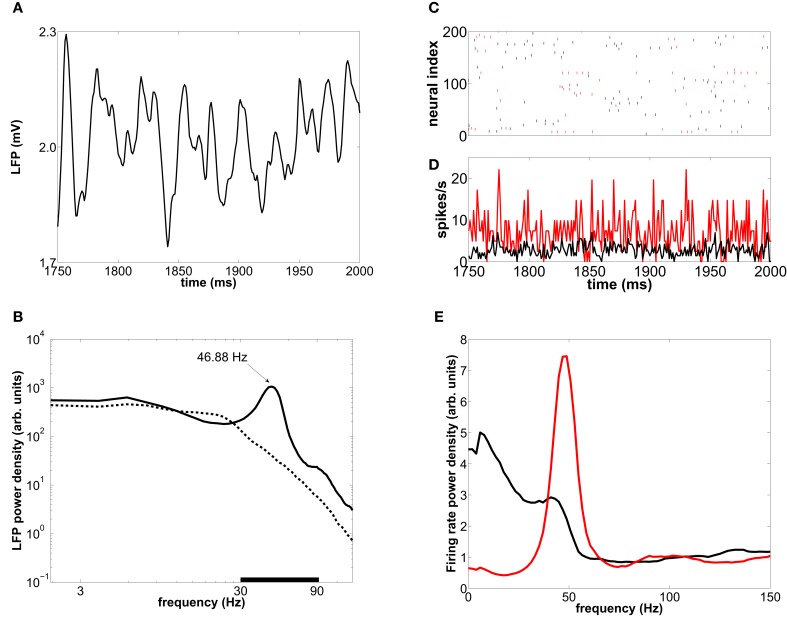
**Collective oscillations in a type-I-neuron network. (A)** LFP time trace in a 250 ms interval for an external mean rate of 8500 spikes/s. **(B)** Averaged LFP power spectrum for an external mean rate of 8500 spikes/s (solid line) and 5000 spikes/s (dashed line). The black bar delimits the gamma-band (30–90 Hz). **(C)** Raster plot of 200 neurons for the same 250-ms interval. **(D)** Time-resolved firing-rate of the whole population. **(E)** The corresponding power spectrum for the excitatory (black) and inhibitory (red) neurons.

When the induced spiking activity is large enough to percolate the network, the internal synaptic currents exhibit a fast cyclic behavior, alternating epochs of high excitation followed by high inhibition, due to the rhythmic synchronization of GABAergic cells via their recurrent connections. The net oscillatory synaptic current leads to a rhythmic behavior in averaged population measures such as the LFP and the time-resolved firing-rate of the population, as shown in Figures 3A,D, respectively. The LFP power spectrum is depicted by solid lines in Figure 3B, revealing a frequency peak whose precise position is determined by the GABAergic synaptic time constants and the synaptic strengths, as well as by the characteristics of the input (Henrie and Shapley, 2005). For a range of biologically plausible values, the frequency of such peak is in the gamma range (30–90 Hz). In particular, for the parameters given in Tables 1, 2 (see section 2), the frequency peak of the LFP spectrum is located around 45 Hz for an external stimulation of mean rate 8500 spikes/s. The low-frequency components are caused by the external incoming spike trains, whose mean rate is an Ornstein–Uhlenbeck process. When the effective coupling between neurons is low, because either the induced spiking activity or the synaptic conductances are weak, the collective rhythm disappears from the LFP power spectrum, as shown by the dashed line in Figure 3B.

Similarly to the LFP, the global firing-rate exhibits a marked peak at ~45 Hz in its spectrum (Figure 3E). This rhythmicity reflects epochs of synchronization between subsets of neurons. Despite the collective rhythmic dynamics (Figure 3D), single neurons display strongly irregular individual spiking (Figure 3C), firing mostly in a sparse and single-spike mode occasionally accompanied by some high-frequency tonic firing, which nevertheless can be compatible with population rhythmic activity at gamma frequencies.

### 3.3. Firing-rate distributions

As described in the last sections there is a striking difference in the spiking activity exhibited by the type I cells once embedded in a network compared to their intrinsic *f-I* curve. To better resolve the firing mode differences and their origin, we first characterize the distribution of instantaneous firing-rates, and then we describe the relation of firing behavior to the input and LFP dynamics.

**Figure 4:**
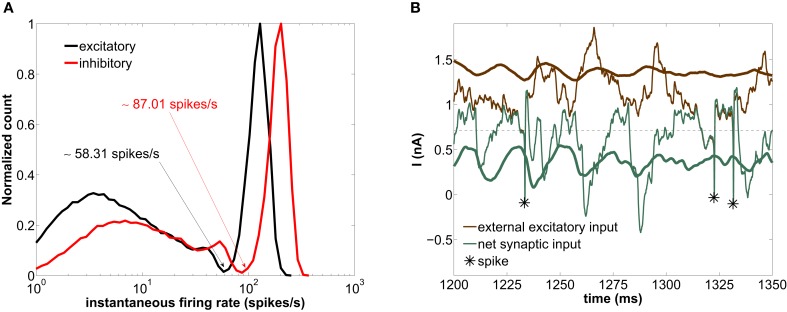
**Modeled network with a GABAergic decay time constant τ_*d*_ = 5 ms. (A)** Histogram of instantaneous firing-rates of both excitatory (black) and inhibitory (red) neurons. The arrows point at the minimum of the distribution separating the fast and slow firing mode. **(B)** Time trace of the external excitatory current (brown lines) and the net synaptic current (green lines) acting upon the excitatory neurons. The thick lines are averages over the entire excitatory population, whereas the thin lines correspond to the current impinging on a single neuron. The asterisks label the spiking times of this neuron. The horizontal dashed line marks the spiking threshold, i.e., the value of *I* at the bifurcation of Figure 1A.

To characterize the distribution of instantaneous firing-rates of individual neurons within the network described in section 2.2, we compute their histogram for the parameter values given in that section, which lead to synchronous irregular firing (Figures 3C,D) with global oscillations in the gamma range (Figures 3B,E). The distribution of instantaneous firing-rates is bimodal (Figure 4A), indicating the prevalence of two firing modes, with high-firing-rate events (short ISIs) reflecting bursts of spiking activity and short-firing-rate events (long ISIs). This effect arises from the network dynamics and cannot be predicted from the characteristic *f-I* curves of type I isolated neurons, which are continuous and hence do not forbid any particular range of firing-rates (Figure 1B; note the gap in the circles, which occurs in network-embedded neurons). This bimodal response was also observed in a network of LIF neurons (Roxin et al., 2011), when the inhibitory population firing-rate exceeded the excitatory population rate. We will address the origin of this bimodal behavior at the end of this section.

The high-firing-rate peak in Figure 4A arises from the spikes of the network-induced bursts (see upper cloud in Figure 1B). The low-firing-rate events are more frequent than the high-firing-rate ones (64% of the interspike intervals of the excitatory population across 20 trials lie below ~58.31 spikes/s—see arrows in Figure 4A). In other words, more neurons are simultaneously found in a silent state or low-firing mode.

In order to understand the genesis of this bimodal firing behavior, we now examine the time evolution of the synaptic currents acting upon the excitatory population (which by definition determines the dynamics of the LFP, see section 2.4). Figure 4B shows the time traces of the external excitatory input (brown lines) and the net synaptic current (green lines). This latter current accounts for both the excitatory component (arising from the external and the recurrent excitatory spikes) and the inhibitory component (arising from the recurrent inhibitory spikes). The thick lines are population averages over the excitatory neurons, whereas the thin lines represent the current values impinging locally on a given neuron. As shown in this figure, the average external synaptic input (thick brown line) lies well above the spiking threshold (horizontal dashed line), whereas the average net synaptic current (thick green line) is below the spiking threshold, due to the inhibitory flow that counteracts the strong external excitation. Therefore, the current impinging on a neuron [thin green line, corresponding to the *I*_syn_ term of Equation (1)] is typically below threshold. Thus neurons spike rarely, only when the excitation-inhibition balance is lost during a certain time window, in which the external input brings the neuron above threshold (see asterisks in Figure 4B). From time to time these intervals are long enough for several spikes to occur in quick succession, giving rise to periods of high-firing-rate (for instance around ~1325 ms). On the other hand, if the excitation-inhibition balance is briefly lost, an isolated spike is elicited only if inhibition is low enough (as we will see in detail in section 3.5), giving rise to low-firing-rate events (for instance at ~1230 ms). Therefore the combined dynamics of excitation and inhibition is the basis of the bimodal distribution of instantaneous firing-rates.

**Figure 5:**
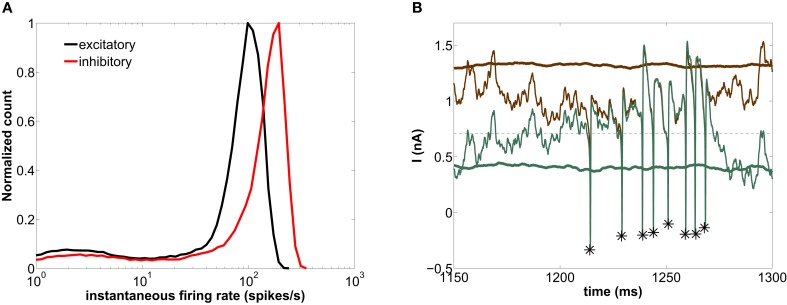
**Modeled network with τ_*d*_ = 30 ms. (A)** Histogram of instantaneous firing-rates of both excitatory (black) and inhibitory (red) neurons. **(B)** Time trace of the external excitatory current (brown lines) and the net synaptic current (green lines) acting upon the excitatory neurons. See caption of Figure 4 for more details on this plot.

According to the previous discussion, the ratio of fast to slow firing events is determined by the characteristic timescale of the periods in which the excitation-inhibition balance is lost. In order to verify this reasoning, we now analyze the behavior of the network for a longer value of the decay time constant τ_*d*_ of the GABAergic synapses. Increasing τ_*d*_ to 30 ms leads to a disappearance of the gamma rhythm (compare the thick green lines in Figures 4B, 5B) (Fisahn et al., 1998; Heistek et al., 2010). Since the inhibitory currents are slower, the periods in which the excitation-inhibition balance is lost are longer, giving rise to an increase in the number of fast spiking events (see asterisks in Figure 5B). This is quantified in Figure 5A, which shows that high firing-rates are much more frequent than low firing ones. Thus the inhibition decay time, τ_*d*_, determines the principal individual firing-rate mode (mostly single-spike for fast inhibition and bursty for slow inhibition).

### 3.4. Coding implications of the firing-rate bimodality

The type I network oscillating in the gamma range, Figure 4A, clearly has a sparse activity, in agreement with the sparse coding of sensory inputs (Olshausen and Field, 2004; Wolfe et al., 2010). We now ask how the bimodality between slow and fast spiking regimes, with a band of forbidden firing-rates, affects the coding capabilities of the neuronal network. To address this question, we establish how the individual firing-rate depends on the external input to the population, rather than on the internal synaptic current (as shown in Figure 1 above). In our case, the external input is modeled by a set of spike trains perturbing each neuron, mimicking either an external sensory input representing a stimulus or neuronal activity arriving from other areas. As mentioned above, the time course of the instantaneous rate is an Ornstein–Uhlenbeck process, equal for all the spike trains (but the specific realization of the Poisson process is different for each neuron). Its mean value is a measure of the input intensity.

**Figure 6:**
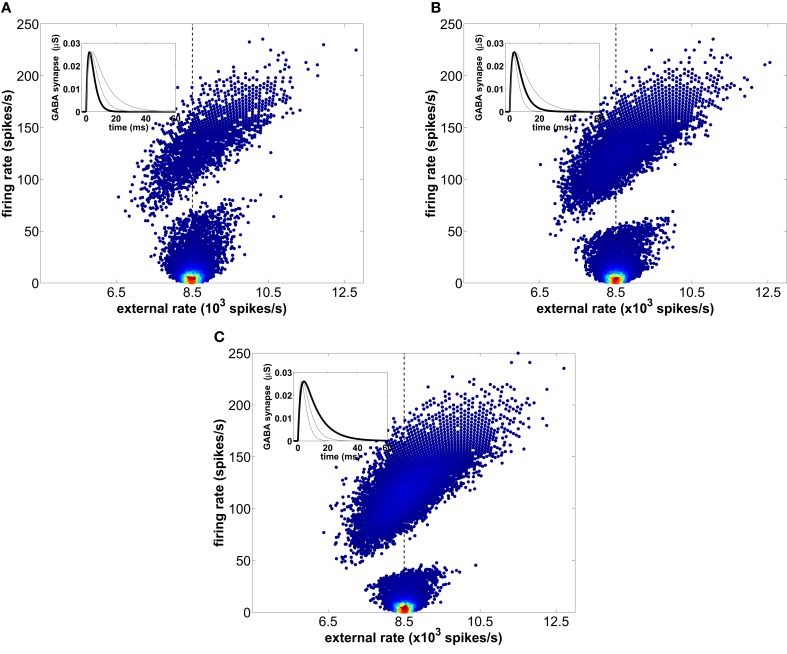
**Instantaneous firing-rates of the excitatory neurons across 20 trials as a function of the mean external firing-rate averaged over each corresponding ISI.** The mean rate of the external spike train is 8500 spikes/s and is marked with a vertical dashed line. Three different time scales of the GABAergic synaptic dynamics, controlled by the decay time constant τ_*d*_, are considered: **(A)** τ_*d*_ = 2.5 ms, **(B)** τ_*d*_ = 5.0 ms (used in the rest of the simulations presented in this study), and **(C)** τ_*d*_ = 10 ms. The local density of firing events within each cloud is represented by a normalized color scale (with red corresponding to high values and dark blue to low values) in order to emphasize that the slow firing mode predominates.

Figure 6 shows the instantaneous individual firing-rate of the excitatory neurons composing the network as a function of the mean external firing-rate, averaged over the corresponding ISI for which the instantaneous firing-rate is calculated, for three different time scales of the GABAergic synaptic dynamics, all of them generating LFP oscillations in the gamma range. All three plots clearly reveal the bimodal character of the firing-rate distribution described above. Two clouds of data points are clearly discernible, corresponding to distinct ISIs. The cloud at the top exhibits a clear correlation with the external rate, which provides for a standard mechanism of rate coding: the higher the external rate is, the faster the resulting firing-rate of the neurons in the network. The lower cloud, on the other hand, is associated with longer firing periods. Naturally, the instantaneous external rate of that low-firing-rate state converges to the mean value of the external rate (marked by a vertical dashed line in the three plots), due to the large periods over which that mean is calculated. Accordingly, for instantaneous external rates clearly above the mean external rate the network only responds with high-frequency firing, following the *f-I* curve of the individual neurons (i.e., operating in a rate coding mode). For moderate external rates, on the other hand, the distribution of firing-rates is bimodal, with low-firing and high-firing events coexisting for the same value of the external rate. This allows the network to encode for the phase of the global oscillations, since in each one of the two modes the locking of the individual spikes to the gamma rhythm is different, as described in section 3.5 below.

The scatter plots shown in Figure 6 also allow us to further investigate the origin of the firing-rate gap evident in those plots (and in Figure 1B above). We can anticipate that the basis of that gap is the fact that due to the recovery time of the network following an increase of inhibition, firing-rates that match the duration of network depression are forbidden. To verify that expectation, we compare the behavior of the network for different values of the decay time constant τ_*d*_ of the GABAergic synapses, while keeping the IPSP amplitude and AMPA synapses constant. Our results show that longer inhibition [growing from panel **(A)** to panel **(C)** in Figure 6] increases the range of forbidden firing periods, because the probability of spiking after an inhibitory barrage is lower during a longer time interval. Accordingly, as τ_*d*_ increases the low-firing mode reduces its area because the slower inhibitory synapses forbid the shortest ISIs within this mode. Therefore, the low-firing state is the result of a competition between the external slow fluctuations and the recurrent inhibition, whose oscillating frequency decreases with increasing GABA synaptic decay time. This leads to the counterintuitive discontinuity in the firing-rate of type I neurons described above. In summary, neurons behave like all-or-none detectors of rapid stimulus fluctuations, with instantaneous firing-rates faster than the LFP gamma peak encoding for the fast dynamics of the stimulus.

### 3.5. Phase locking to the gamma cycle

We have seen that, even though the firing-rate of the individual neurons is far from being tonic, the probability of firing across the network varies rhythmically in time with a frequency around 45 Hz. This rhythm is generated by recurrent excitatory and inhibitory connections, and is revealed in the LFP and firing-rate dynamics. Both the synaptic flow (apparent in the LFP) and the spiking activity are mutually interacting, given that a decrease in synaptic inhibition triggers an increase in the mean firing activity of the network. This causal relationship implies that the peaks of the population firing activity and the troughs of the LFP are displaced in time within the millisecond range, controlled by the synaptic delay.

To further characterize the effect of the global rhythm on the firing activity, we have computed STA across trials of both the LFP and the inhibitory synaptic current, *I*_GABA_, acting upon each neuron. The LFP carries information about the global synaptic activity affecting the excitatory neurons of the population (see Equation 2), and is therefore a measure of the global activity of the network. In contrast, *I*_GABA_ only accounts for the inhibitory synaptic current impinging on an neuron from the firing activity of its presynaptic inhibitory neurons, and is thus a local measure. We have considered the STA in a time window of 70 ms around a spike (50 ms pre-spike and 20 ms post-spike). Furthermore, to avoid having a previous spike from the same neuron fall within the time window being considered, we have only taken into account spikes at least 50 ms apart. When computing the STA we have classified the spikes, according to the bimodal distribution of instantaneous firing-rates shown in Figure 4A, as slow or fast firing events, the latter corresponding to network-induced bursts. Within the fast firing mode we selected the first spike of each burst, since we are interested in the events leading to burst initiation. We now discuss separately the mechanisms underlying the generation of the slow and fast firing modes and their relationship with the phase of the global oscillations.

#### 3.5.1. Fast firing mode

**Figure 7:**
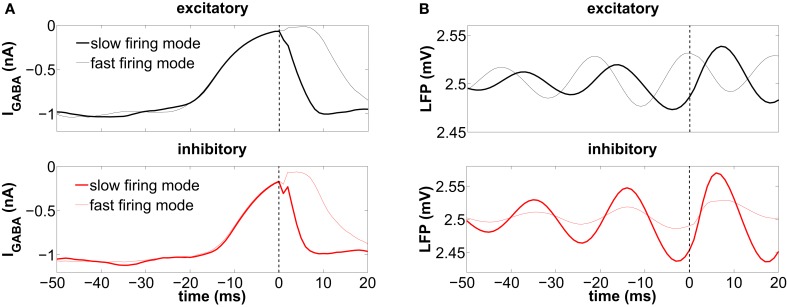
**Spike-triggered averages (STA) from (A) the synaptic inhibitory current impinging each individual cell and (B) the LFP.** Spikes from ISIs larger than 50 ms are considered. The thick lines correspond to those spikes prior to a long ISI (slow firing mode), while the thin lines correspond to the short ISIs (fast firing mode). These plots are obtained from a single trial, and the frontier between long and short ISIs is set at 17.14 ms (58.31 spikes/s) for the excitatory neurons and at 11.49 ms (87.01 spikes/s) for the inhibitory neurons. The vertical dashed line is the reference time at which a spike is elicited.

As shown by the thin lines in Figure 7A, the high-frequency regime appears when the inhibitory synaptic current *I*_GABA_ impinging on a neuron (i.e., its *local* inhibition) is close to zero for a relatively large time interval right after *t* = 0. In other words, the presynaptic inhibitory neurons must be silent for a long enough period of time after the neuron fires, in order for a burst to be initiated. In those conditions, it is more probable that the excitatory external input brings the neuron above the spiking threshold for a sustained amount of time, giving rise to a fast spiking period.

**Figure 8:**
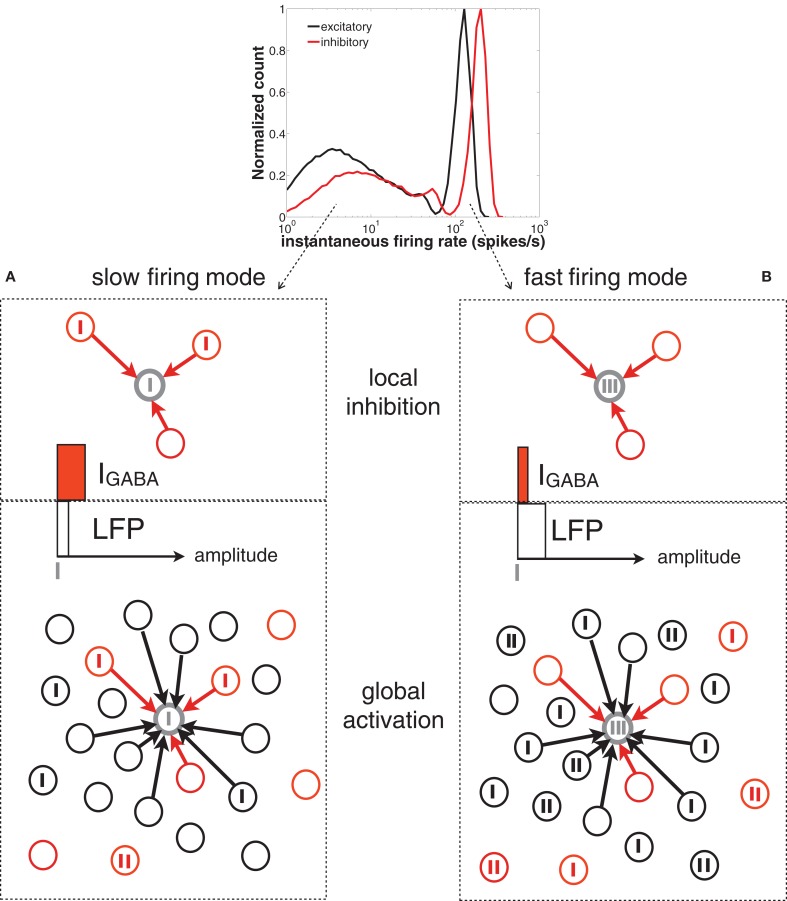
**Scheme of the network spiking activity underlying slow (A) and fast (B) firing modes of a given neuron in the network (represented by the gray circle).** Vertical thick ticks represent spikes. The black and red circles represent excitatory and inhibitory neurons, respectively. For simplicity, only contacts to the gray neuron are represented (black arrows). In the top panel of **(A)** (slow firing mode), the reference neuron (in gray) is surrounded by presynaptic inhibitory neurons that are starting to fire (i.e., the level of *I*_GABA_, represented by a horizontal red bar, is moderate). The global activity of the network (bottom panel, represented by a white horizontal bar) is low and the earliest inhibitory spikes prevent the cells from bursting. In the top panel of **(B)** (fast firing mode) the neuron is surrounded by inactive inhibitory neurons (i.e., the level of *I*_GABA_ is low), whose silence is driven by the high activity of the rest of the inhibitory population (reflected in a high level of the LFP).

For the excitatory neurons this occurs preferentially at a high level of *global* inhibition, i.e., at the maxima of the LFP (thin line in the top panel of Figure 7B). These two events, a maximum of the global inhibition (i.e., of the LFP) and a minimum of the local inhibition (i.e., of |*I*_GABA_|), occur simultaneously, since it is when global inhibition is strong that some neurons can be locally surrounded by strongly inactivated inhibitory neurons. During this time interval, those neurons fire in bursts due to the external excitation, before all the inhibitory neurons become excited and prevent the firing of the rest of the population. A scheme of the interplay between local inhibition and global activation leading to the fast firing mode is shown in Figure 8B. In contrast with the excitatory neurons, in inhibitory neurons the initiation of the fast firing mode does not depend so strongly on the level of global inhibition (thin line in the bottom panel of Figure 7B). We hypothesize that these neurons, which have a smaller membrane time constant than the excitatory ones (see section 2), react rapidly to the external excitatory fluctuations and spike regardless of the level of global inhibition, provided their presynaptic inhibitory current is zero (thin line in the bottom panel of Figure 7A).

#### 3.5.2. Slow firing mode

The low-frequency regime, on the other hand, takes place when the local inhibitory current, *I*_GABA_, impinging on the neuron is momentarily zero, as shown by the thick line in Figure 7A. The rapid increase of inhibition following a spike (at *t* = 0, dashed line) prevents the neuron from spiking again. This happens at low levels of global inhibition, i.e., close to the minima of the LFP (thick line in Figure 7B). Immediately after a spike the firing activity of the population increases, and thus the local inhibition grows as well, increasing the distance of individual neurons to threshold (see the scheme of Figure 8A).

**Figure 9:**
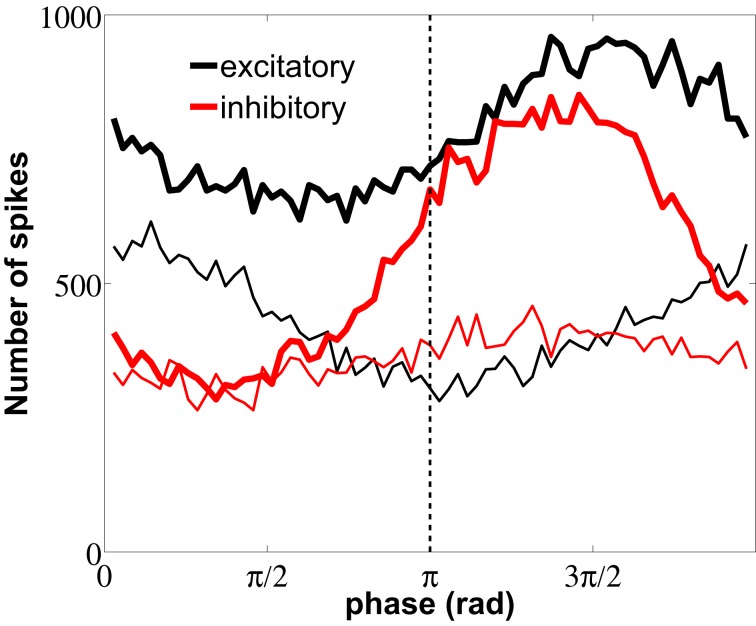
**Distribution of LFP phase values at which neurons spike.** The plot distinguishes between spikes flanking ISIs with fast (thin lines) and slow (thick lines) firing modes, for both excitatory (black) and inhibitory (red) neurons. Measures were performed over the same data used in Figure 7. The dashed line marks the π phase that corresponds to the LFP minimum.

The STA analysis discussed above shows that the simultaneous occurrence of slow instantaneous rates at both the excitatory and inhibitory populations (thick lines in Figure 7B) is only possible near the minima of the LFP, whereas fast instantaneous rates can only be simultaneously present in both populations at the maxima of the LFP (thin lines in Figure 7B). Given this interaction between the individual firing modes and the rhythmic network dynamics, one can ask whether a partial representation of the input is coded in the spiking timing of cells relative to the phase of the population oscillation. We now address this question by proceeding to filter the LFP signal around the gamma frequency peak (46.88 ± 5 Hz) and assign an instantaneous phase to the LFP time series via the analytical signal approach (Hilbert phase) (Le Van Quyen et al., 2001). A histogram of the LFP phase values at which the neurons spike is shown in Figure 9, with π corresponding to the LFP troughs (vertical dashed line in the figure).

Spike firings are widely distributed over the 2π cycle of the LFP phase because, as seen from the raster plot of Figure 3C, the firing activity is noisy and the correlation between neurons is weak, due to their low coupling and the absence of a common drive. We have performed the spike-LFP phase locking analysis for both high-firing (thin lines) and low-firing (thick lines) events, taking only into account the first spikes of short and long ISIs, respectively. Figure 9 shows that the slow firing regime of the inhibitory neurons (red thick line) is more sensitive to the LFP phase than the fast firing regime, resulting in a more pronounced locking to the troughs of the LFP, with a delay ≥2 ms due to the mean synaptic delay. The high-firing mode of the excitatory neurons, on the other hand, is phase-locked with no delay to the LFP peaks around 0 (note that no synaptic delay is considered in the external train of spikes, which as mentioned above controls the high-firing regime).

In section 3.4 we have shown that the fast fluctuations of the excitatory external rate induce high instantaneous firing-rates, and thus this mode encodes the fast dynamics of the input. In this section we have additionally shown that the occurrence of spikes relative to the LFP phase depends on the firing mode. In particular, the high-frequency mode appears with higher probability when inhibition is maximal (the LFP phase is 0) and the low-frequency mode appears when inhibition is minimal (at the rising LFP phases near π). This constitutes a mechanism of phase coding that complements the rate-coding mechanism depicted in Figure 6, according to which fast external fluctuations were unambiguously encoded by a high-frequency bursting activity of the neurons.

## 4. Discussion

In this paper we have studied the firing characteristics of a neural network composed of type I neurons. From a single-neuron perspective, both the *f-I* and PRC have been widely studied and related to the kind of bifurcation that takes the cells from rest to tonic firing. On the other hand, it is known that cortical neurons, wired among them in a complex manner, do not spike periodically but rather sparsely and randomly. The *f-I* curve of the excitatory and inhibitory neural types helps to determine how the synaptic connection strength has to be tuned in order to balance excitation with inhibition, in agreement with the firing-rates of each population. Moreover, according to their phase response, type I neurons tend to fire faster within the network than in isolation.

The recurrent connections between the excitatory and inhibitory population lead to rhythmicity in the synaptic current impinging on a neuron, composed by a succession of a flood of excitation and a flood of inhibition. These oscillations are a collective phenomenon arising from the interactions among neurons, and their frequency can be slowed down with increasing GABA synaptic duration. In that way, gamma oscillations in the firing-rate activity and LFP signal arise naturally from the recurrent connections between excitatory and inhibitory neurons, when the synaptic conductances are adjusted to balance excitation with inhibition.

As long as inhibitory neurons fire more intensely than the excitatory ones to compensate for their relative small number, the network settles in a non-periodic firing state with a significant gamma-band component. At frequencies closer or below the gamma LFP peak the external drive is balanced by inhibition, which leads to a low-firing mode that arises separated from the more “natural” fast-firing mode (given by the single-neuron *f-I* curve) by a gap of quasi-forbidden instantaneous firing-rates. This leads to a discontinuity in the firing-rates of the neurons, similar to what happens in single type II neurons but that does not occur in type I neurons in isolation. Thus, this firing-rate discontinuity constitutes an emerging dynamical property of the network.

Although the code strategy used by cortical neurons is still a matter of debate, bimodal distributions of instantaneous firing-rates are found experimentally in both the auditory cortex (Shih et al., 2011) and the visual cortex (DeBusk et al., 1997; Snider et al., 1998). Other modeling works have also shown a bimodal distribution of ISIs (Wang, 1998) using intrinsically bursting neurons, in contrast with our case in which the bimodality appears naturally from the balanced network as in (Roxin et al., 2011). Moreover, in Bereshpolova et al. (2011) the recorded single unit activity through different awake brain states, shows that during alert periods some particular neural types reduce their bursting with respect to the non-alert periods. In agreement with our results, the alert state corresponds to higher power in the gamma range.

In conclusion, the balance of an excitatory synaptic current by a strong inhibitory current yields to a discontinuity in the firing-rate of individual neurons forming a neural network. The highest instantaneous rates encode fast fluctuations of the external stimulus, while the spiking times of the network occurring at moderate fluctuations of the input with respect to the mean encode the phase of the LFP. Therefore neural networks can efficiently implement two coding strategies: (1) a rate code for the fast bursting mode, sensitive to rapid changes in the processing of stimuli and (2) a phase code for the slower input fluctuations, according to which isolated spikes occur at the troughs of the LFP, whereas bursts of frequency higher than the gamma peak of the global oscillations arise at the peaks of the LFP. This second feature might contribute to an internal cortical representation of the input. In summary, these results show that cortical population activity depends non-trivially on the dynamical properties of the underlying neurons, and that global population measures shape the firing dynamics of the constituent cells, allowing for multiple encoding mechanisms to be implemented in networks with balanced excitation and inhibition.

### Conflict of interest statement

The authors declare that the research was conducted in the absence of any commercial or financial relationships that could be construed as a potential conflict of interest.

## References

[B1] Berens P. Logothetis N. K. Tolias A. S. (2010). Local field potentials, bold and spiking activity – relationships and physiological mechanisms. Nat. Precedings. Available online at: http://hdl.handle.net/10101/npre.2010.5216.1

[B2] Bereshpolova Y. Stoelzel C. R. Zhuang J. Amitai Y. Alonso J.-M. Swadlow H. A. (2011). Getting drowsy? alert/nonalert transitions and visual thalamocortical network dynamics. J. Neurosci. 31, 17480–17487 10.1523/JNEUROSCI.2262-11.2011 22131409 PMC6623815

[B3] Bokil H. Andrews P. Kulkarni J. E. Mehta S. Mitra P. P. (2010). Chronux: a platform for analyzing neural signals. J. Neurosci. Methods 192, 146–151 10.1016/j.jneumeth.2010.06.020 20637804 PMC2934871

[B4] Brunel N. (2000). Dynamics of sparsely connected networks of excitatory and inhibitory spiking neurons. J. Comput. Neurosci. 8, 183–208 10809012 10.1023/a:1008925309027

[B5] Buzsáki G. Anastassiou C. A. Koch C. (2012). The origin of extracellular fields and currents – EEG, ECoG, LFP and spikes. Nat. Rev. Neurosci. 13, 407–420 10.1038/nrn3241 22595786 PMC4907333

[B6] Buzsáki G. Draguhn A. (2004). Neuronal oscillations in cortical networks. Science 304, 1926–1929 10.1126/science.1099745 15218136

[B7] DeBusk B. C. DeBruyn E. J. Snider R. K. Kabara J. F. Bonds A. B. (1997). Stimulus-dependent modulation of spike burst length in cat striate cortical cells. J. Neurophysiol. 78, 199–213 9242274 10.1152/jn.1997.78.1.199

[B8] El Boustani S. Pospischil M. Rudolph-Lilith M. Destexhe A. (2007). Activated cortical states: experiments, analyses and models. J. Physiol. 101, 99–109 10.1016/j.jphysparis.2007.10.001 18023562

[B9] Ermentrout B. (2002). Simulating, Analyzing, and Animating Dynamical Systems: A Guide to XPPAUT for Researchers and Students, Vol. 56. Philadelphia, PA: SIAM

[B10] Fisahn A. Pike F. G. Buhl E. H. Paulsen O. (1998). Cholinergic induction of network oscillations at 40 Hz in the hippocampus *in vitro*. Nature 394, 186–189 10.1038/28179 9671302

[B11] Fries P. (2009). Neuronal gamma-band synchronization as a fundamental process in cortical computation. Annu. Rev. Neurosci. 32, 209–224 10.1146/annurev.neuro.051508.135603 19400723

[B12] Garcia-Ojalvo J. Sancho J. M. (1999). Noise in Spatially Extended Systems. New York, NY: Springer-Verlag 10.1103/physreve.49.2769 9961542

[B13] Gutfreund Y. Yarom Y. Segev I. (1995). Subthreshold oscillations and resonant frequency in guinea-pig cortical neurons: physiology and modelling. J. Physiol. 483, 621–640 7776248 10.1113/jphysiol.1995.sp020611 PMC1157807

[B14] Hansel D. Mato G. (2003). Asynchronous states and the emergence of synchrony in large networks of interacting excitatory and inhibitory neurons. Neural Comput. 15, 1–56 10.1162/089976603321043685 12590818

[B15] Hansel D. Mato G. Meunier C. (1995). Synchrony in excitatory neural networks. Neural Comput. 7, 307–337 8974733 10.1162/neco.1995.7.2.307

[B16] Heistek T. S. Jaap Timmerman A. Spijker S. Brussaard A. B. Mansvelder H. D. (2010). GABAergic synapse properties may explain genetic variation in hippocampal network oscillations in mice. Front Cell Neurosci. 4:18 10.3389/fncel.2010.00018 21082021 PMC2901093

[B17] Henrie J. A. Shapley R. (2005). LFP power spectra in V1 cortex: the graded effect of stimulus contrast. J. Neurophysiol. 94, 479–490 10.1152/jn.00919.2004 15703230

[B18] Izhikevich E. M. (2007). Dynamical Systems in Neuroscience. Computational Neuroscience. Cambridge: MIT press

[B19] Le Van Quyen M. Foucher J. Lachaux J. Rodriguez E. Lutz A. Martinerie J. (2001). Comparison of hilbert transform and wavelet methods for the analysis of neuronal synchrony. J. Neurosci. Methods 111, 83–98 11595276 10.1016/s0165-0270(01)00372-7

[B20] Markram H. Toledo-Rodriguez M. Wang Y. Gupta A. Silberberg G. Wu C. (2004). Interneurons of the neocortical inhibitory system. Nat. Rev. Neurosci. 5, 793–807 10.1038/nrn1519 15378039

[B21] Mazzoni A. Panzeri S. Logothetis N. K. Brunel N. (2008). Encoding of naturalistic stimuli by local field potential spectra in networks of excitatory and inhibitory neurons. PLoS Comput. Biol. 4:e1000239 10.1371/journal.pcbi.1000239 19079571 PMC2585056

[B22] Olshausen B. A. Field D. J. (2004). Sparse coding of sensory inputs. Curr. Opin. Neurobiol. 14, 481–487 10.1016/j.conb.2004.07.007 15321069

[B23] Rinzel J. Ermentrout G. B. (1989). Analysis of Neural Excitability and Oscillations. Methods in Neuronal Modeling. Cambridge: MIT Press

[B24] Roxin A. Brunel N. Hansel D. Mongillo G. van Vreeswijk C. (2011). On the distribution of firing rates in networks of cortical neurons. J. Neurosci. 31, 16217–16226 10.1523/JNEUROSCI.1677-11.2011 22072673 PMC6633220

[B25] Rué P. Garcia-Ojalvo J. (2011). Gene circuit designs for noisy excitable dynamics. Math. Biosci. 231, 90–97 10.1016/j.mbs.2011.02.013 21419138

[B26] Shih J. Y. Atencio C. A. Schreiner C. E. (2011). Improved stimulus representation by short interspike intervals in primary auditory cortex. J. Neurophysiol. 105, 1908–1917 10.1152/jn.01055.2010 21307320 PMC3075280

[B27] Shinomoto S. Miura K. Koyama S. (2005). A measure of local variation of inter-spike intervals. Bio. Syst. 79, 67–72 10.1016/j.biosystems.2004.09.023 15649590

[B28] Shinomoto S. Sakai Y. Funahashi S. (1999). The ornstein-uhlenbeck process does not reproduce spiking statistics of neurons in prefrontal cortex. Neural Comput. 11, 935–951 10226190 10.1162/089976699300016511

[B29] Singer W. (1999). Neuronal synchrony: a versatile code for the definition of relations? Neuron 24, 49–65 10.1016/S0896-6273(00)80821-1 10677026

[B30] Snider R. K. Kabara J. F. Roig B. R. Bonds A. B. (1998). Burst firing and modulation of functional connectivity in cat striate cortex. J. Neurophysiol. 80, 730–744 9705464 10.1152/jn.1998.80.2.730

[B31] Softky W. R. Koch C. (1993). The highly irregular firing of cortical cells is inconsistent with temporal integration of random epsps. J. Neurosci. 13, 334–350 8423479 10.1523/JNEUROSCI.13-01-00334.1993 PMC6576320

[B32] Soriano J. Rodríguez Martínez M. Tlusty T. Moses E. (2008). Development of input connections in neural cultures. Proc. Natl. Acad. Sci. U.S.A. 105, 13758–13763 10.1073/pnas.0707492105 18772389 PMC2544527

[B33] Thomson D. J. (1982). Spectrum estimation and harmonic analysis. Proc. IEEE 70, 1055–1096

[B34] van Vreeswijk C. Sompolinsky H. (1996). Chaos in neuronal networks with balanced excitatory and inhibitory activity. Science 274, 1724–1726 10.1126/science.274.5293.1724 8939866

[B35] Wang X.-J. (1998). Calcium coding and adaptive temporal computation in cortical pyramidal neurons. J. Neurophysiol. 79, 1549–1566 9497431 10.1152/jn.1998.79.3.1549

[B36] Wang X.-J. Buzsáki G. (1996). Gamma oscillations by synaptic inhibition in a hippocampal interneuronal network. J. Neurosci. 16, 6402–6413 8815919 10.1523/JNEUROSCI.16-20-06402.1996 PMC6578902

[B37] Wolfe J. Houweling A. R. Brecht M. (2010). Sparse and powerful cortical spikes. Curr. Opin. Neurobiol. 20, 306–312 10.1016/j.conb.2010.03.006 20400290

